# The Functional and Anatomical Impacts of Healthy Muscle Ageing

**DOI:** 10.3390/biology12101357

**Published:** 2023-10-23

**Authors:** James P. Charles, Karl T. Bates

**Affiliations:** Department of Musculoskeletal & Ageing Science, Institute of Life Course and Medical Sciences, University of Liverpool, Liverpool L7 8TX, UK; k.t.bates@liverpool.ac.uk

**Keywords:** diffusion tensor imaging, magnetic resonance imaging, muscle architecture

## Abstract

**Simple Summary:**

Ageing sometimes leads to changes in the size, shape and structure of muscles. This impacts their ability to perform their functions and so leads to an increased likelihood of injuries and negatively impacts quality of life. This work attempts to investigate how the structure and performance of individual muscles of the human leg may change due to ageing, using a combination of magnetic resonance imaging and muscle strength testing in groups of aged (65+ years old) and young (18–40 years old) individuals. The results showed a larger difference in muscle structure and strength in the muscles that extend the knee (i.e., the quadriceps) between the age groups relative to other muscles, and these muscles also showed the strongest relationships between structure and strength. There was also greater variation in fibre lengths and variables linked to fibre type and quality within individual muscles in the aged group compared to the young group. Overall, these results show that even healthy ageing may cause large changes in muscle structure and decreases in performance and that the quadriceps muscles could be a target for exercises to reduce the negative effects of ageing.

**Abstract:**

Even “healthy” muscle ageing is often associated with substantial changes in muscle form and function and can lead to increased injury risks and significant negative impacts on quality of life. However, the impacts of healthy muscle ageing on the fibre architecture and microstructure of different muscles and muscle groups throughout the lower limb, and how these are related to their functional capabilities, are not fully understood. Here, a previously established framework of magnetic resonance and diffusion tensor imaging was used to measure the muscle volumes, intramuscular fat, fibre lengths and physiological cross-sectional areas of 12 lower limb muscles in a cohort of healthily aged individuals, which were compared to the same data from a young population. Maximum muscle forces were also measured from an isokinetic dynamometer. The more substantial interpopulation differences in architecture and functional performance were located within the knee extensor muscles, while the aged muscles were also more heterogeneous in muscle fibre type and atrophy. The relationships between architecture and muscle strength were also more significant in the knee extensors compared to other functional groups. These data highlight the importance of the knee extensors as a potential focus for interventions to negate the impacts of muscle ageing.

## 1. Introduction

Muscle ageing is commonly associated with changes in muscle structure (i.e., architecture [1]). For example, decreases in muscle fibre lengths and muscle volumes, as well as increases in intramuscular fat, have been noted previously, which all ultimately contribute to reduced force output with age. While severe cases of muscle ageing can be diagnosed as sarcopenia [2,3,4,5], even healthy ageing (i.e., not clinically sarcopenic) can result in a loss of muscle strength [6] and negative impacts on quality of life, such as through increased risks of falling [7,8] or various lower limb musculoskeletal injuries [9,10]. However, the impacts of even healthy muscle ageing on the architecture of different muscles and muscle groups throughout the lower limb, and how these are related to their functional capabilities, are not comprehensively understood.

Studying the architecture and microstructure of individual muscles can be facilitated by the use of diffusion tensor imaging (DTI), which images soft tissue based on the relative diffusion of water along its three principal axes, each quantified through eigenvalues (λ1, λ2 and λ3). This method has become an established method by which to visualise and quantify individual skeletal muscle architecture [11,12,13], and subsequently accurately characterise and quantify muscle function [14]. This method has also revealed the degree to which various lower limb muscles, such as soleus [15] and semitendinosus [14], exhibit substantial heterogeneity (or compartmentalisation) in fibre lengths throughout the muscle belly, which is likely to be functionally important [16]. But other variables are obtainable from diffusion tensor images, including various indices quantifying the water diffusion throughout each tissue such as fractional anisotropy (FA—the ratio of the three eigenvalues) and mean diffusivity (MD—the average of the three eigenvalues). These indices provide information regarding the microstructure and physiological capabilities, where FA has been shown to be positively associated, and MD negatively associated, with the abundance of type I (slow twitch) fibres within a muscle [17]. FA may also be positively related to the degree of fibre atrophy and the amount of extracellular tissue (i.e., fat) within a muscle [18].

Previous work using DTI to study muscle ageing has shown higher FA, shorter fibre lengths and an increase in the heterogeneity of muscle fibre lengths in the gastrocnemius muscle of aged relative to younger individuals [18], as well as the rectus femoris and biceps femoris muscles of the proximal lower limb [19]. While the somewhat limited selection of muscles sampled for these studies precludes any conclusions regarding how muscle ageing may impact functional performance throughout the lower limb, studies investigating muscle degeneration following strokes [20] and prolonged sedentary periods (e.g., space flight [21]) have shown that situations similar to ageing may disproportionately affect certain lower limb muscle groups such as the knee extensors and ankle plantarflexors. Ultimately, a detailed investigation into how ageing differentially impacts individual muscles and functional groups throughout the lower limb is needed in order to truly assess the impacts of muscle ageing on daily life.

Thus, the aims of this work are to (1) compare the muscle architecture and microstructure of 12 lower limb muscles between young and healthily aged populations, (2) quantify the within-muscle heterogeneity of these metrics, and (3) relate variations in these to variations in maximum muscle force output. Studying these parameters on an individual muscle as well as functional group level will allow for more insight into how even healthy ageing impacts muscle functional performance, and ultimately inform interventions to negate the impacts of muscle ageing.

## 2. Methods

Ten aged individuals (mean ± standard deviation: age—72.1 ± 4.61 years; body mass—73.6 ± 11.1 kg; BMI—24.5 ± 3.30 kgm^−2^; Table S1) were recruited for this study via the Aughton and Ormskirk U3A (https://aughton-ormskirk-u3a.co.uk/). Each participant completed a SARC-F questionnaire prior to their participation [22], with no individual showing any clinical signs of sarcopenia or other musculoskeletal conditions (and were thus considered healthily aged; Table S2). The 10 young individuals (age—27.2 ± 3.68 years; body mass 71.4 ± 8.92 kg; BMI 22.9 ± 2.09 kgm^−2^), who provided a comparison to the aged individuals, were recruited as part of a prior study [11,23].

### 2.1. Isokinetic Testing

Maximum muscle functional performance of the right-side knee extensor/flexors and ankle plantarflexors/dorsiflexors was tested through isometric (joint positions: knee extension—60° extension; knee flexion—0°; ankle plantarflexion—0°; ankle dorsiflexion—25° plantarflexion) exercises using an isokinetic dynamometer (HUMAC NORM, CSMi, Stoughton, MA, USA). Each isometric repetition lasted 5 s with 5 s rest between each repetition, for a total of 5 repetitions. Each subject was given verbal encouragement during each trial to ensure they exerted maximum torque. These conditions are the same as those described by Charles et al. [11] and Charles et al. [23]. The maximum isometric torques (Nm) from each repetition were averaged and then normalised to body weight and lower limb length to generate single dimensionless values for maximum isometric torque for each individual.

### 2.2. MR Imaging and Analysis

From each subject (aged and young), 12 muscles in the right lower limb of each were analysed here: knee flexors: biceps femoris—long head (BFL), biceps femoris—short head (BFS), semitendinosus (ST), semimembranosus (SM); knee extensors: rectus femoris (RF), vastus lateralis (VL), vastus medialis (VM), vastus intermedius (VI); ankle plantarflexors: medial gastrocnemius (MG), lateral gastrocnemius (LG), soleus (SOL); ankle dorsiflexors: tibialis anterior (TA). From each muscle, the following metrics were measured: fibre length (mm, normalised to body mass^0.33^), fibre pennation angle, intramuscular fat (% of total muscle volume), “effective” muscle volume (total muscle volume—intramuscular fat), physiological cross-sectional area (PCSA, mm^2^, normalised to body mass^0.66^), fractional anisotropy and mean diffusivity (×10^−3^ mm^2^s^−1^).

**Figure 1:**
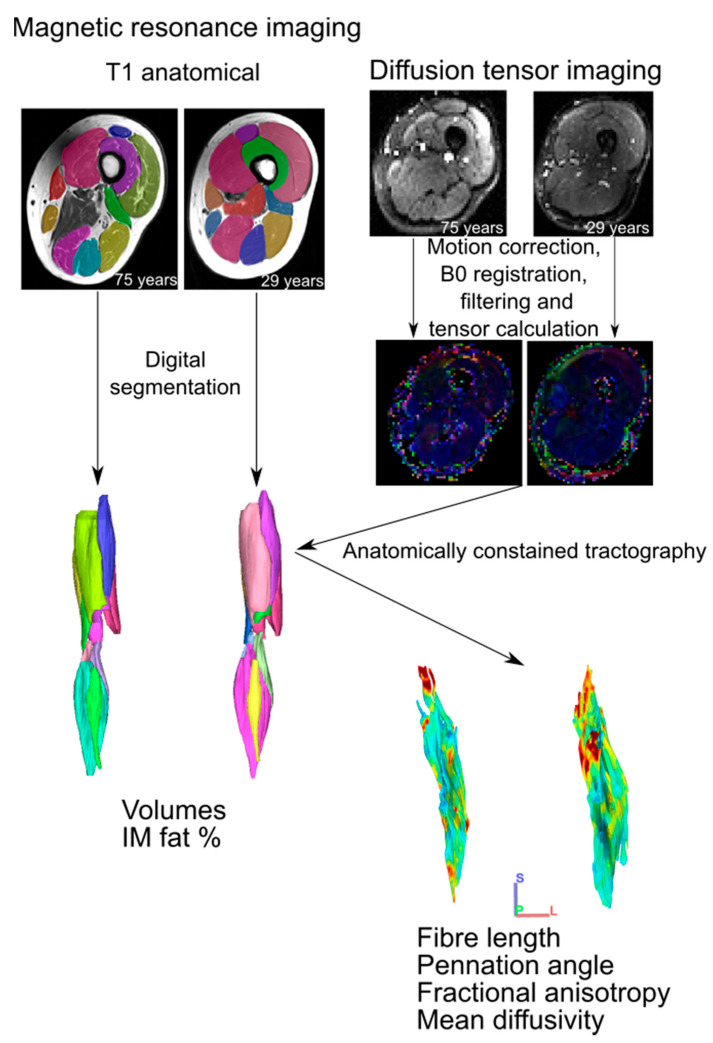
Magnetic resonance imaging analysis workflow. For each participant, T1 anatomical images were digitally segmented to generate 3D individual muscle meshes, from which muscle volume and intramuscular fat % could be measured. Diffusion tensor images were preprocessed prior to tensor calculation, from which muscle fibre tracts were generated via deterministic fibre tractography. These raw fibre tracts were truncated to the boundaries of their corresponding 3D mesh through anatomically constrained tractography [13] to generate predictions of muscle fibre length, pennation angle, fractional anisotropy and mean diffusivity. These steps follow those established previously [11].

The detailed framework for the acquisition and processing of the MR and DT images to obtain the metrics described above has been described previously [11] and is summarised in Figure 1. In short, two sequences were used to image both lower limbs of each subject, from the proximal surface of iliac crest to the distal surface of the foot: (1) T1: weighted anatomical TSE (voxel size 0.4395 × 0.4395 × 6.5 mm^3^; repetition time (TR): 700 ms; echo time (TE): 28 ms; number of slices: 36 per segment; number of signal averages (NSA): 1) and (2) diffusion-weighted single-shot dual-refocusing spin-echo planar (voxel size 2.96 × 2.96 × 6.5 mm^3^; TR/TE 7900/67 ms; 12 direction diffusion gradients; *b* value: 0 and 400 s/mm^2^; strong fat suppression: spectral attenuated inversion recovery (SPAIR); number of slices: 36 per segment; NSA: 1; bandwidth: 2350 Hz/pixel). Total scanning time was ~45 min. The T1 anatomical images were used to derive measurements of muscle volumes and intramuscular fat through digital segmentation in Mimics software v25 (Materialise, Leuven, Belgium), while the DT images were used to measure fibre architecture (fibre lengths and pennation angles) and microstructure (FA and MD) using deterministic fibre tractography in DSI studio software v2022.08.03 [24]. The DT images were B0 corrected, motion corrected and filtered using the same steps outlined in (REF). Median fibre length and pennation angle measurements were subsequently obtained using “anatomically constrained tractography”, where raw fibre tracts for each muscle were truncated to terminate at the boundaries of their corresponding 3D volumetric mesh [13]. From the muscle volumes, fibre lengths and pennation angles, the physiological cross-sectional area (PCSA) of each muscle was calculated:

$$\mathrm{PCSA}=(V_{m}\;\times \;\cos\theta )/(L_{f})$$

where *V_m_* is muscle (belly) volume (mm^3^), *L_f_* is muscle fibre length (mm), *θ* is muscle fibre pennation angle.

### 2.3. Muscle Heterogeneity

The heterogeneity of L_f_, FA and MD within each muscle was quantified through the calculation of the coefficient of variation, skewness and bimodal coefficient [25].

Coefficient of variation (CV):

$$\mathrm{CV}=\frac{\mathrm{SD}}{\bar{x}}$$

where SD is the standard deviation and $\bar{x}$ is the mean value with each population.

Skewness (*S*):

$$S=\frac{n}{\left(n-1\right)\left(n-2\right)}\sum {\left(\frac{x_{i}-\bar{x}}{SD}\right)}^{3}$$

where *n* is the number of muscle fibres. A skewness value > 0.5 suggests a positive skew to the distribution of fibre lengths within a muscle, and a value < −0.5 suggests a negative skew.

Bimodal coefficient (*b*):

$$b=\frac{S^{2}+1}{k+\frac{3{\left(n-1\right)}^{2}}{\left(n-2\right)\left(n-3\right)}}\;$$

where k is the excess kurtosis. A bimodal coefficient >0.555 suggests the fibres within a muscle have a bimodal distribution.

### 2.4. Statistical Analyses

The normality of each metric was tested using Shapiro–Wilk tests. One-way analyses of variance (ANOVA) were used to test for statistically significant different differences between muscle architecture, microstructure and muscle strength between the aged and young populations. Linear regressions were used to test for relationships between log-normalised muscle architecture and muscle strength within the entire study population (aged + young). All statistical tests were carried out in OriginPro Version 2016 (OriginLab Corporation, Northampton, MA, USA).

## 3. Results

### 3.1. Isokinetic Testing

**Figure 2:**
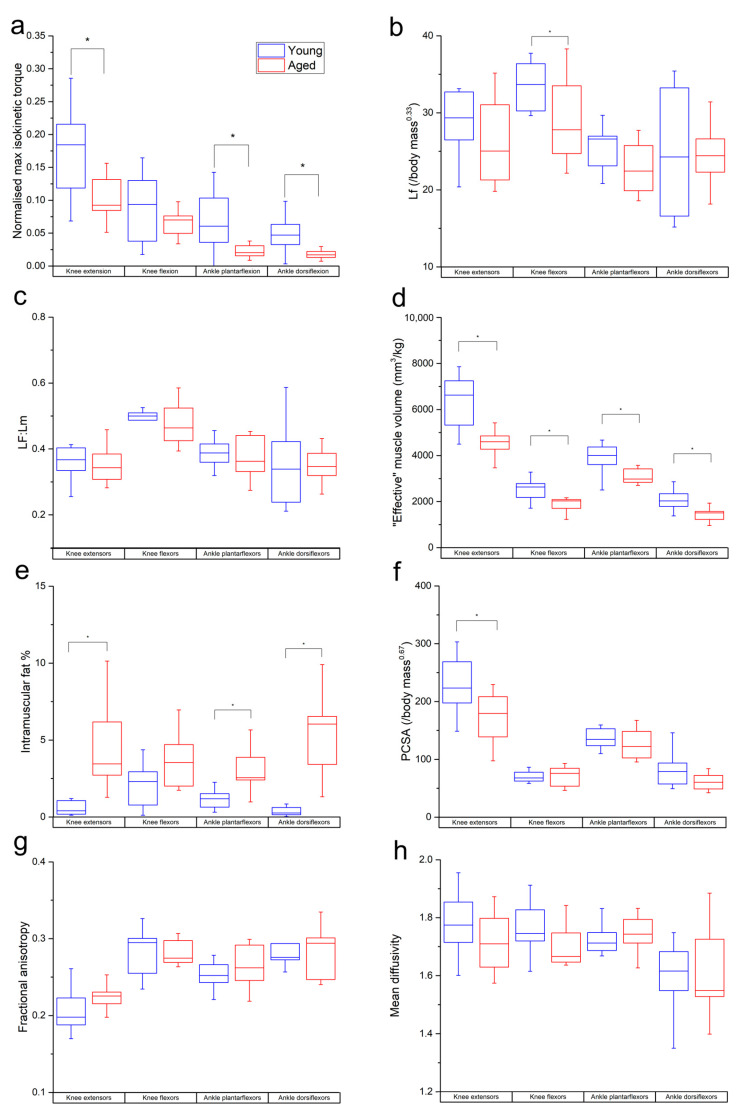
Differences in normalised maximum isometric muscle torque (**a**), muscle fibre length (**b**), L_f_:L_m_ (**c**), muscle volume (normalised to body mass) (**d**), PCSA (**e**), intramuscular fat % (**f**), fractional anisotropy (**g**) and mean diffusivity (**h**) between the young and aged populations. Values are averaged over each muscle within each functional group. * indicates statistically significant differences (*p* < 0.05). On each box, the central horizontal line indicates the median value and the bottom and top edges of the box indicate the 25th and 75th percentiles. Whiskers extend to the most extreme data points.

The aged population showed a decrease in maximum isometric torque relative to the young population (Figure 2a) during knee extension (young: 0.23 ± 0.07; aged: 0.16 ± 0.03), knee flexion (young: 0.14 ± 0.04; aged: 0.11 ± 0.03), ankle plantarflexion (young: 0.11 ± 0.05; aged: 0.06 ± 0.02) and ankle dorsiflexion (young: 0.046 ± 0.01; aged: 0.043 ± 0.01). These differences were statistically significant in knee extension (*p* = 0.01) and ankle plantarflexion (*p* = 0.01), but not knee flexion (*p* = 0.17) or ankle dorsiflexion (*p* = 0.60).

### 3.2. Muscle Architecture

The muscle architecture data of individual subjects are listed in Tables S3–S22, while population averages and *p* values for interpopulation comparisons are shown in Table S23. The differences in individual muscle architecture between the age groups are shown in Figure S1.

L_f_ and L_f_:L_m_ were lower in the age population than the young population in most individual muscles and functional groups (Figure S1a,b and Figure 2b,c). However, these differences were only statistically significant in BFL (*p* = 0.02), VI (*p* = 0.02), LG (*p* = 0.04) and on a functional group level in the knee flexors in L_f_ (young: 33.7 ± 3.05 mm/kg^0.33^; aged: 29.3 ± 5.75 mm/kg^0.33^; *p* = 0.04).

“Effective” muscle volumes were significantly lower in the aged population relative to the young population in most muscles (other than BFS, MG and SOL) and all functional groups (knee extensors: young: 6335 ± 1215 mm^3^kg^−1^, aged: 4417 ± 776 mm/kg^0.33^, *p* = <0.01; knee flexors: young: 2534 ± 480 mm^3^kg^−1^, aged: 1965 ± 384 mm^3^kg^−1^, *p* = <0.01; ankle plantarflexors: young: 3880 ± 678 mm^3^kg^−1^, aged: 3198 ± 586 mm^3^kg^−1^, *p* = 0.02; ankle dorsiflexors: young: 2046 ± 457 mm^3^kg^−1^, aged: 1515 ± 416 mm^3^kg^−1^, *p* = 0.01; Figure 2d). IM fat % was significantly higher in all muscles (except SM, ST and LG; Figure S1d) and all muscle groups except the knee flexors (knee extensors: young: 0.55 ± 0.42%, aged: 4.48 ± 2.63, *p* = <0.01; ankle plantarflexors: young: 1.21 ± 0.66, aged: 2.97 ± 1.34, *p* = <0.01; ankle dorsiflexors: young: 0.36 ± 0.29%, aged: 5.18 ± 2.61%, *p* = <0.01; Figure 2e). PCSA was lower in the aged population in most muscles; however, this was only statistically significant in VI, VL and VM (Figure S1e), as well as most functional groups (except knee flexors). However, this was only statistically significant in the knee extensors (young: 226 ± 46, aged: 174 ± 42, *p* = 0.01; Figure 2f).

In the functional groups, there were nonsignificant trends for FA to be higher (knee extensors: young: 0.20 ± 0.02, aged: 0.22 ± 0.01; ankle plantarflexors: young: 0.25 ± 0.01, aged: 0.26 ± 0.02; Figure 2g) and for MD to be lower (knee extensors: young: 1.75 ± 0.15 × 10^−3^ mm^2^s^−1^, aged: 0.71 ± 0.10 × 10^−3^ mm^2^s^−1^; knee flexors: young: 1.76 ± 0.09 × 10^−3^ mm^2^s^−1^, aged: 1.69 ± 0.06 × 10^−3^ mm^2^s^−1^; ankle dorsiflexors: young: 1.6 ± 0.11 × 10^−3^ mm^2^s^−1^, aged: 1.61 ± 0.15 × 10^−3^ mm^2^s^−1^; Figure 2h) in the aged population compared to the young. Within individual muscles, there was a significantly higher FA in MG (*p* = 0.03; Figure S1f) and lower MD in SM (*p* = 0.02) and ST (*p* = 0.01; Figure S1g) in the aged population.

**Figure 3:**
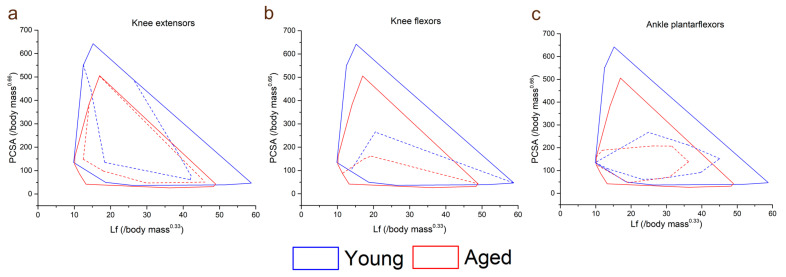
Total lower limb muscle functional morphospace (solid lines) compared to that of the knee extensors (**a**), knee flexors (**b**) and ankle plantarflexors (**c**) (dashed lines) in the young and aged populations. Here, muscles can be classed as force specialised (high PCSA, low fibre length), displacement specialised (high fibre length, low PCSA) or power specialised (high PCSA, high fibre length). In the aged population, the total functional morphospace is smaller and characterised by lower PCSAs and fibre lengths. However, the knee extensors take up a larger proportion of the entire lower limb muscle functional morphospace in the aged population than in the young population, suggesting a lack of functional specialisation in these muscles with increasing age. The shapes and relative sizes of the knee flexor and ankle plantarflexor muscles are similar between the age groups.

The functional specialisations of the muscles within each functional group were visualised using functional morphospace plots (Figure 3). Here, the total functional morphospace of the 12 lower limb muscles studied is smaller in the aged population than the young, where there are more muscles with apparent specialisation for high force output (high PCSA, low L_f_) and high velocity (high L_f_, low PCSA). In the young population, the morphospace of the knee extensors (Figure 3a) is smaller relative to the entire morphospace compared to the aged population; however, these proportions are similar between the age groups in the knee flexors (Figure 3b) and ankle plantarflexors (Figure 3c).

### 3.3. Within-Muscle Heterogeneity

**Figure 4:**
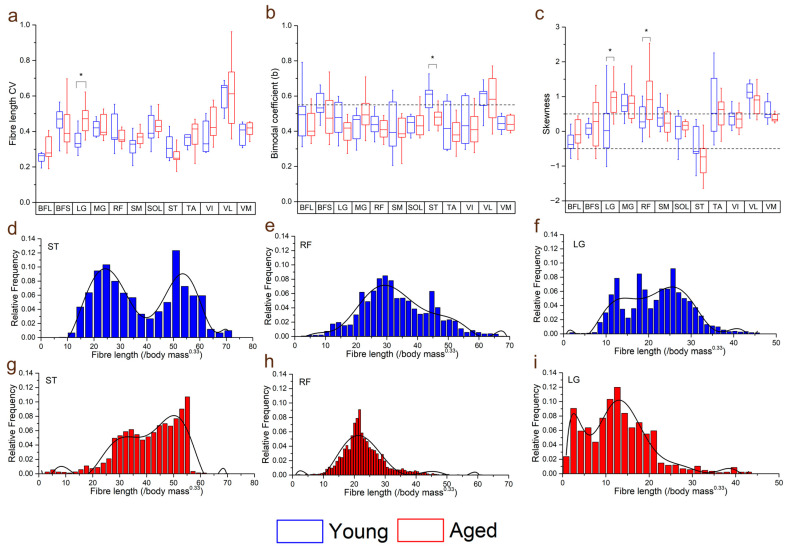
Differences in the coefficients of variation (CV: averaged over each muscle within each functional group) (**a**), bimodal coefficient (**b**) and skewness (**c**) between the young and aged populations. Muscles with a bimodal coefficient >0.55 can be classed as having a bimodal distribution of fibre lengths. Muscles with skewness values ≥0.5 can be classed as positively skewed, and ≤−0.5 as negatively skewed. Representative fibre length distributions in the young (**d**–**f**) and aged (**g**–**i**) populations are also shown, where semitendinosus (ST) had, on average, a bimodal distribution in the young population, but not in the aged. Rectus femoris (RF) and lateral gastrocnemius (LG) were both positively skewed in the aged population, with a higher proportion of shorter fibres, while these distributions were less skewed in the young population. For muscle definitions, see Methods. * indicates statistically significant differences (*p* < 0.05). On each box, the central horizontal line indicates the median value and the bottom and top edges of the box indicate the 25th and 75th percentiles. Whiskers extend to the most extreme data points. Ninth-order polynomial functions were used to approximate the fibre length distributions of each muscle (**d**–**i**).

Within individual muscles, the CV of L_f_ in general increased in the aged population compared to the young population (Figure 4). However, this difference was only statistically significant in LG (young: 0.35 ± 0.06, aged: 0.46 ± 0.08, *p* = <0.01; Figure 4a). When averaged over functional groups, CVs were generally higher in the aged population in most functional groups (except the knee flexors) with the only significant difference seen in the ankle plantarflexors sas (young: 0.39 ± 0.03, aged: 0.44 ± 0.05, *p* = 0.01; Figure S2a). The distributions of these fibre lengths (skew and bimodality) within individual muscles were not significantly different in most muscles, other than ST, which was on average classed as bimodally distributed in the young population, but not the aged (young: 0.57 ± 0.10, aged: 0.47 ± 0.05, *p* = 0.01; Figure 4b,d,g) and LG and RF, which were on average significantly more positively skewed in the aged population (LG: young: 0.14 ± 0.84, aged: 0.90 ± 0.51, *p* = 0.02; RF: young: 0.37 ± 0.41, aged: 1.05 ± 0.91, *p* = 0.04; Figure 4c,e,f,h,i).

**Figure 5:**
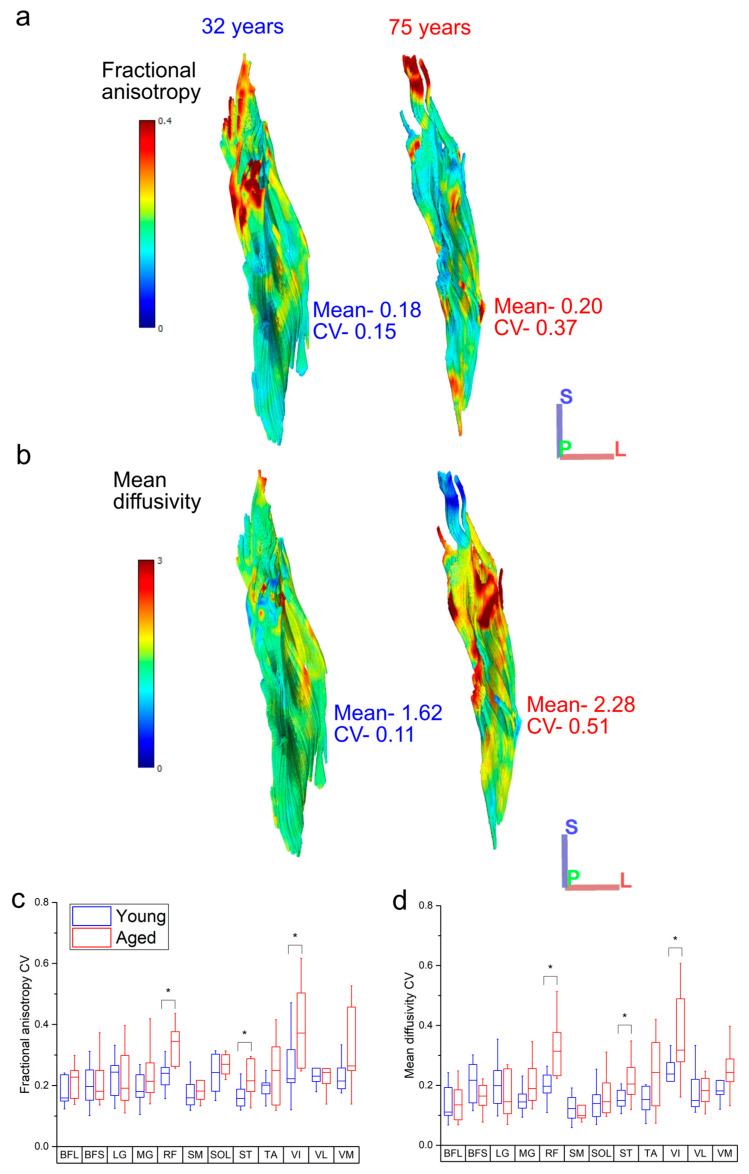
Representative heat maps of the distribution of fractional anisotropy (FA) (**a**) and mean diffusivity (MD) (**b**) in the rectus femoris of one young and one aged individual. The aged muscle shows increased heterogeneity in both metrics, particularly in the distal portions of muscle, which shows increased regionalisation. When averaged over each functional group, the knee extensors rectus femoris (RF) and vastus intermedius (VI) show significantly more variation in both FA (**c**) and MD (**d**) in the aged population relative to the young population. For muscle definitions, see Methods. * indicates statistically significant differences (*p* < 0.05). On each box, the central horizontal line indicates the median value and the bottom and top edges of the box indicate the 25th and 75th percentiles. Whiskers extend to the most extreme data points. Axis definition: S = superior; L = lateral; P = posterior.

Variations in both FA and MD were also generally higher in the aged population across all muscles (Figure 5c,d), with statistically significant differences in the CVs of both metrics seen in RF (FA: young: 0.25 ± 0.09, aged: 0.34 ± 0.06, *p* = 0.05; MD: young: 0.21 ± 0.07, aged: ^0.33^ ± 0.1, *p* = <0.01), ST (FA: young: 0.16 ± 0.03, aged: 0.22 ± 0.06, *p* = 0.02; MD: young: 0.15 ± 0.03, aged: 0.22 ± 0.07, *p* = 0.02) and VI (FA: young: 0.26 ± 0.10, aged: 0.39 ± 0.12, *p* = 0.02; MD: young: 0.23 ± 0.07, aged: 0.41 ± 0.24, *p* = 0.03). Within each functional group, this variation was only significantly higher in the knee extensors (FA: young: 0.25 ± 0.08, aged: 0.32 ± 0.05, *p* = 0.03; MD: young: 0.20 ± 0.04, aged: 0.28 ± 0.08, *p* = 0.01; Figure S2b,c).

### 3.4. Muscle Architecture vs. Muscle Strength

**Figure 6:**
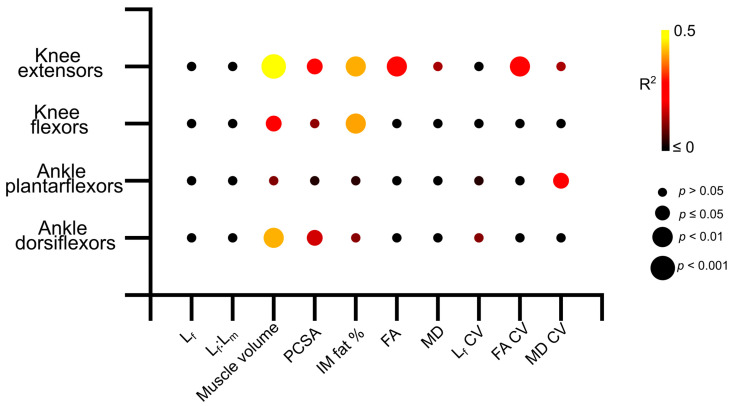
Matrix showing the R^2^ values and statistical significance of the linear relationships between log-normalised maximum isometric muscle torque and muscle architecture and microstructure averaged over each muscle within each functional group across the entire study population (aged and young participants). The knee extensors show stronger relationships between architecture and strength than other muscle groups. L_f_—fibre length. L_f_:L_m_—fibre length muscle length ratio. PCSA—physiological cross-sectional area. IM fat %—intramuscular fat %. FA—fractional anisotropy. MD—mean diffusivity.

Knee extension isometric torques were significantly positively related to knee extensor muscle volume (R^2^ = 0.47, *p* = <0.01; Figure 6 and Figure S3b) and PCSA (R^2^ = 0.28, *p* = 0.01; Figure 6 and Figure S3c), and negatively related to IM fat (R^2^ = 0.41, *p* = <0.01; Figure 6 and Figure S3d), mean FA (R^2^ = 0.27, *p* = 0.01; Figure 6 and Figure S3e) and FA CV (R^2^ = 0.29, *p* = 0.01; Figure 6 and Figure S7e). In the knee flexors, there were significant positive relationships between muscle torque and knee flexor muscle volume (R^2^ = 0.262, *p* = 0.01; Figure 6 and Figure S4b) and negative relationships to IM fat (R^2^ = 0.40, *p* =< 0.01; Figure 6 and Figure S4d). Ankle plantarflexion isometric torques were only significantly negatively related to the variation of MD within the ankle plantarflexor muscles (R^2^ = 0.25, *p* = 0.02; Figure 6, Figures S5 and S7k), while ankle dorsiflexion isometric torques were significantly positively related to muscle volume (R^2^ = 0.41, *p* =< 0.01; Figure 6 and Figure S6b) and PCSA (R^2^ = 0.17, *p* = 0.03; Figure 6 and Figure S6d). Full linear regression statistics are shown in Table S24. The results of the Shapiro–Wilk test for normality on each of these variables are shown in Tables S25–S27.

## 4. Discussion

The aim of this study was to quantify the differences in individual muscle architecture and microstructure between a healthily aged population and a young, healthy population, and relate these variations to maximum muscle functional performance. While the diffusion tensor imaging framework has been used previously to study the anatomical and functional impacts of healthy ageing [18,19], this is the first study to investigate these interactions in multiple functional groups throughout the lower limb as well as study the potential impacts of fibre architecture heterogeneity on functional performance.

In terms of muscle functional performance, muscle strength clearly reduced on the whole in the aged population relative to the young population (Figure 2a). However, these deficits were more substantial and statistically significant in the knee extensors and ankle plantarflexors, which are the high-force-producing “anti-gravity” muscles that play important roles in daily locomotor tasks such as walking [26] and the sit–stand transition [27]. These large differences in muscle strength capacity are therefore indicative of important functional deficits in even healthily aged individuals and are reflective of known decreases in locomotor performance due to ageing [28].

These muscle strength deficits in the aged population are somewhat mirrored in the differences in muscle architecture, microstructure and heterogeneity seen between the study populations (Figure 2, Figure 3, Figure 4 and Figure 5). In general, the aged population showed decreases in fibre lengths, muscle volumes, PCSA and mean diffusivity, although the decreases in fibre lengths and PCSA, which are commonly associated with a muscle’s force-generating capacity, were less significant than those seen in muscle strength. These are similar to findings reported by Goodpaster et al. [29] and Delmonico et al. [30], who attributed this discrepancy to a more substantial decrease in muscle quality than quantifiable force-generating capacity, which is reflected in the increases in intramuscular fat % and fractional anisotropy seen here in the aged population. These differences, while not surprising, support previous work that has shown significant intramuscular fat infiltrations [31] and overall shifts towards a higher relative abundance of slower, type I muscle fibres [32,33,34] associated with muscle atrophy. However, the degree of these differences varied between individual muscles (Figure S1) and functional groups. For instance, large and statistically significant differences were seen primarily in the knee extensors, in terms of volume, PCSA and intramuscular fat %, while these differences (volume and PCSA in particular) were less notable in the other functional groups (Figure 2). However, the significantly shorter fibre lengths in the knee flexors may have helped them “maintain” a consistent PCSA with age despite significantly lower muscle volumes. There was also a substantial difference in the functional specialisations of the knee extensors between the age groups, shown by visualising the functional morphospace occupied by these muscles relative to the overall lower limb muscle morphospace [14,35] (Figure 3). These plots highlight specific anatomical functional specialisations of muscles within a single muscle functional group based on the relationships between their fibre length and PCSA, or force-generating capacity, with muscles with short fibres and high PCSAs being adapted for high force production and those with long fibres and low PCSAs being adapted for high contraction velocities. Here, the knee extensors of the young individuals appear to occupy less of the overall lower limb morphospace than those of the aged individuals, suggesting that these muscles may lose their anatomical specialisations for high force production with age. This, however, does not appear to extend to other muscle functional groups, with these differences being much less substantial in the knee flexors and ankle plantarflexors, despite a trend for shorter knee flexor fibre lengths in the aged population.

Furthermore, there appeared to be a general increase in the variation, or heterogeneity, of fibre architecture and microstructure in the aged compared to the young population (Figure 4 and Figure S2). For instance, the variations in fibre length were significantly higher in the ankle plantarflexors (particularly LG) (Figure 3a), and there were also some substantial differences in how these fibre lengths were distributed throughout the muscles. The lengths of the ST fibres, for example, were classed as bimodal in the young population (on average), but normally distributed in the aged population (Figure 3d,g), which is reflective of the known compartmentalisation of this muscle [36] and the possible subsequent loss of this with age. Furthermore, both RF and LG were significantly more positively skewed in the aged population in terms of fibre length, with a greater tendency for shorter fibres in these muscles compared to the young individuals. Significant differences were also seen between the heterogeneity of FA, associated with increased fibre atrophy and an increase in type I muscle fibres [17,18], and MD, negatively associated with an increase in type I muscle fibres [17], within the RF, VI and ST muscles (Figure 5c, d). So, while the overall difference in these metrics between the age groups was not statistically significant (Figure 2g,h), despite general trends for FA to increase and MD to decrease with age, healthy ageing does appear to introduce significant heterogeneity to functionally important muscles in terms of both fibre type and atrophy, as well as (to a lesser and less consistent extent) fibre length.

These differences in muscle architecture and microstructure between the young and aged populations, as well as larger degrees of heterogeneity in these metrics seen in the aged individuals, appear to be functionally important. Significant relationships were found between knee extensor isometric torque and various aspects of gross architecture such as muscle volume, intramuscular fat and PCSA, as well as FA and the variation (or heterogeneity) of this in the knee extensors (Figure 6, Figures S3 and S7). However, these relationships were less clear within the other muscle functional groups, despite similar decreases in maximum isometric torque seen in the ankle plantarflexors in the aged individuals, with only the variation in MD showing significant relationships to plantarflexor strength (Figure 6, Figure S7k). These relationships, along with those seen in the knee flexors and ankle dorsiflexors, not only confirm direct correlations between age-related changes in muscle architecture, particularly reductions in muscle volume and infiltrations of intramuscular fat, and deficits in muscle functional performance, but also highlight the potential important of the knee extensors in maintaining overall functional performance with increasing age. Given the importance of proximal lower limb muscles for producing power and work during even steady-state locomotion [37,38,39,40,41], as well as in response to changes in substrate compliance [42], it is reasonable to suggest that interventions to prevent the negative effects of even healthy ageing should focus on maintaining the volume and overall force-generating capacity in the knee extensors.

The higher degree of potential heterogeneity in muscle fibre types and atrophy seen here in the aged population relative to the young are also potentially functionally important, particularly when considering experimental determinations of muscle function in a biomechanical context. For instance, when recording muscle activity using electromyography [42], or predicting muscle forces and work outputs using biomechanical models and simulations [43], it is standard practice to consider each muscle as a single functional unit. However, the use of high-density EMG arrays [44,45] have shown that various muscles in both the upper and lower limb can contain a large number of functionally distinct units. And, in the context of biomechanical modelling specifically, representing muscles with multiple actuators (i.e., distinct functional units) has been shown to significantly impact predictions of muscle–tendon function [46]. It is therefore likely that separating muscles into distinct functional regions is necessary to truly determine the functional impacts of muscle ageing or other neuromuscular conditions.

## 5. Conclusions

These results show significant differences in muscle architecture, particularly muscle volumes, intramuscular fat and PCSA, in healthily aged individuals compared to a younger population. There were also significant decreases in strength output from most muscle function groups in the aged population. The knee extensor muscles showed the most significant anatomical changes in healthy ageing, which appear to be significantly related to the negative impacts on functional performance. The MR imaging and tractography approach means that these assessments of muscle architecture are based on a relatively large number of fibre measurements within each subject, which previous work has shown to be crucial to accurate representations of the gross anatomical of individual muscles [14]. However, the relatively expensive and time-consuming nature of this approach means that the sample size in terms of number of individuals (i.e., *n* = 10 per cohort) is somewhat limited in this study, which may limit statistical power and/or the wider applicability of our results overall. Nevertheless, this work represents a solid foundation for future work utilizing DTI to study the functional impacts of muscle ageing, potentially in a larger cohort.

## Data Availability

All MR images used to obtain estimates of muscle architecture are available from the University of Liverpool’s Research Data Catalogue (DataCat; datacat.liverpool.ac.uk/id/eprint/2500).

## Supplementary Materials

The following supporting information can be downloaded at: https://www.mdpi.com/article/10.3390/biology12101357/s1.
