# Supplementary material for: The Functional and Anatomical Impacts of Healthy Muscle Ageing
Source: Biology (Basel). 2023 Oct 23;12(10):1357. doi: 10.3390/biology12101357 (PMC10604714; doi:10.3390/biology12101357)
Supplement: Supplementary file 1 [file biology-12-01357-s001.zip › ESM_final.pdf]

Supplementary Material for: The functional and anatomical impacts of healthy muscle ageing

James P Charles* & Karl T Bates

Department of Musculoskeletal & Ageing Science, Institute of Life Course and Medical Sciences, University of Liverpool, Liverpool, UK; k.t.bates@liverpool.ac.uk

*Corresponding author E-mail: j.charles@liverpool.ac.uk

Contains:

Supplementary figures

Supplementary tables (Provided within "Supplementary tables.xlsx")

Supplementary table captions:

Table S1- Subject demographics. Young participants are the same as those used by Charles et al. [1]

Table S2- Pooled results from the SARC-F questionnaire for diagnosing sarcopenia.

Tables S3-22- Muscle architecture data from aged (Tables S3-12) and young (13-22) individuals

Table S23- Averaged muscle architecture over the aged and young study populations (n=10 per group), and the statistical significance ($p \leq 0.05$) of the inter-population comparisons of each variable.

Table S24- Linear regression statistics for relationships between normalised maximum isometric torque and various aspects of muscle architecture within the entire study population (10 young + 10 aged). Statistically significant relationships are bold and italicised.

Table S25- Results of a Shapiro-Wilk test for normality on each variable included in the linear regression analysis. A p-value <0.05 suggests a non-normal distribution.

Table S26- Results of a Shapiro-Wilk test for normality on each variable included in the Young group. A p-value <0.05 suggests a non-normal distribution.

Table S27- Results of a Shapiro-Wilk test for normality on each variable included in the Aged group. A p-value <0.05 suggests a non-normal distribution.

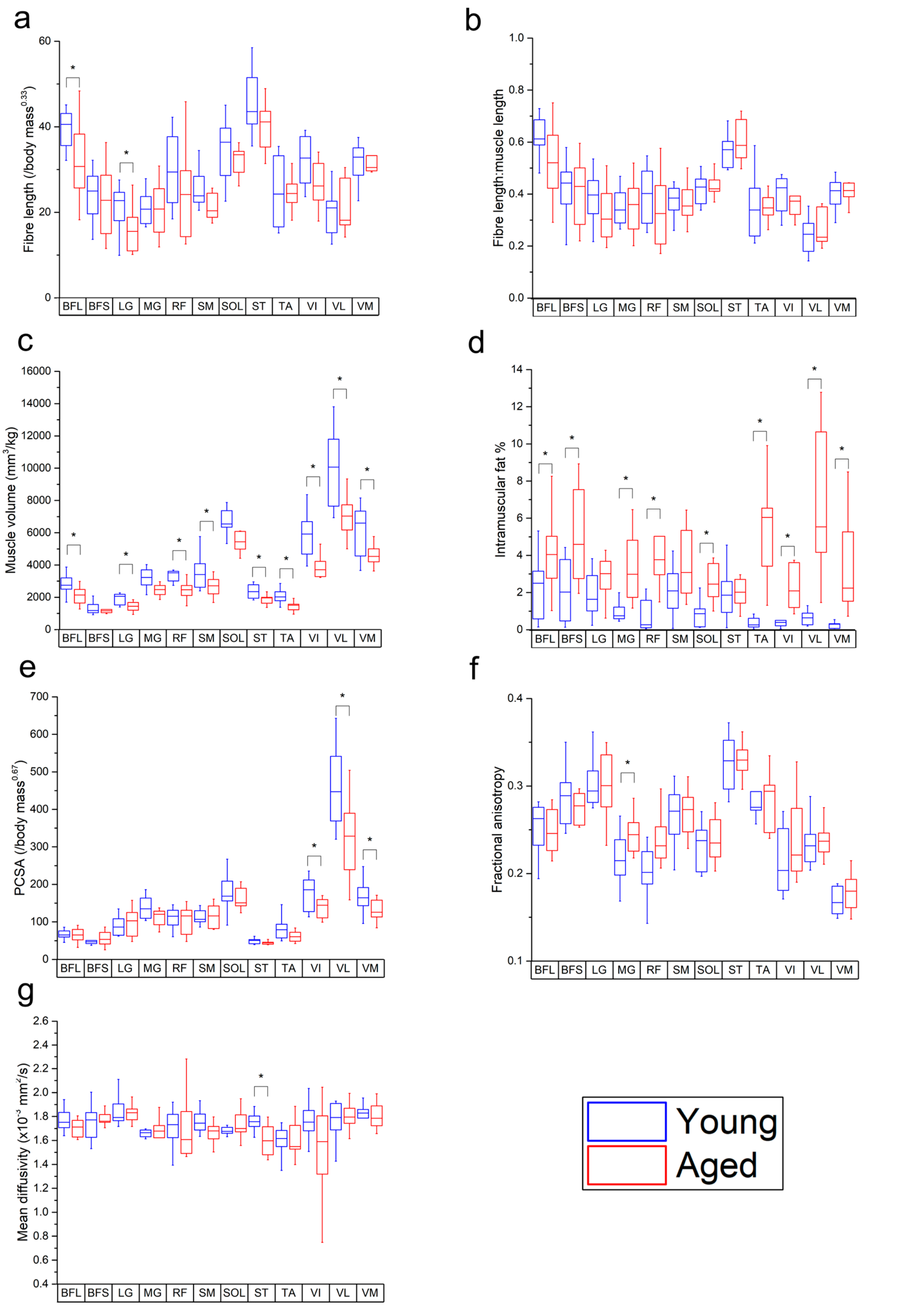

Figure S1. Differences in individual muscle fibre length (a), fibre length:muscle length (b), muscle volume (c), intramuscular fat % (d), physiological cross sectional area (e), fractional anisotropy (f) and mean diffusivity (g) between the young and aged groups. * indicates statistically significant differences ($p < 0.05$).

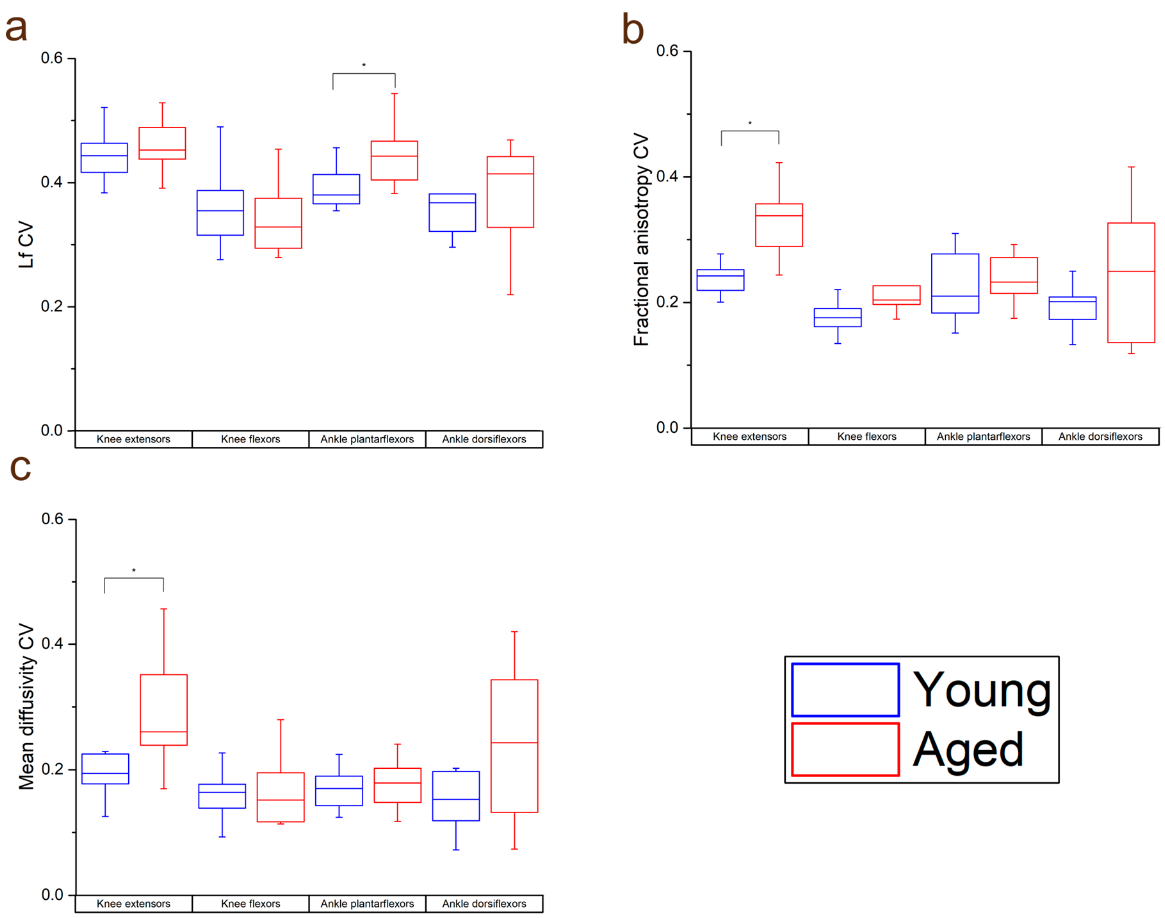

Figure S2. The variation, or heterogeneity, in functional group averages in fibre lengths (a), fractional anisotropy (b) and mean diffusivity (c) between the young and aged groups. * indicates statistically significant differences ($p < 0.05$).

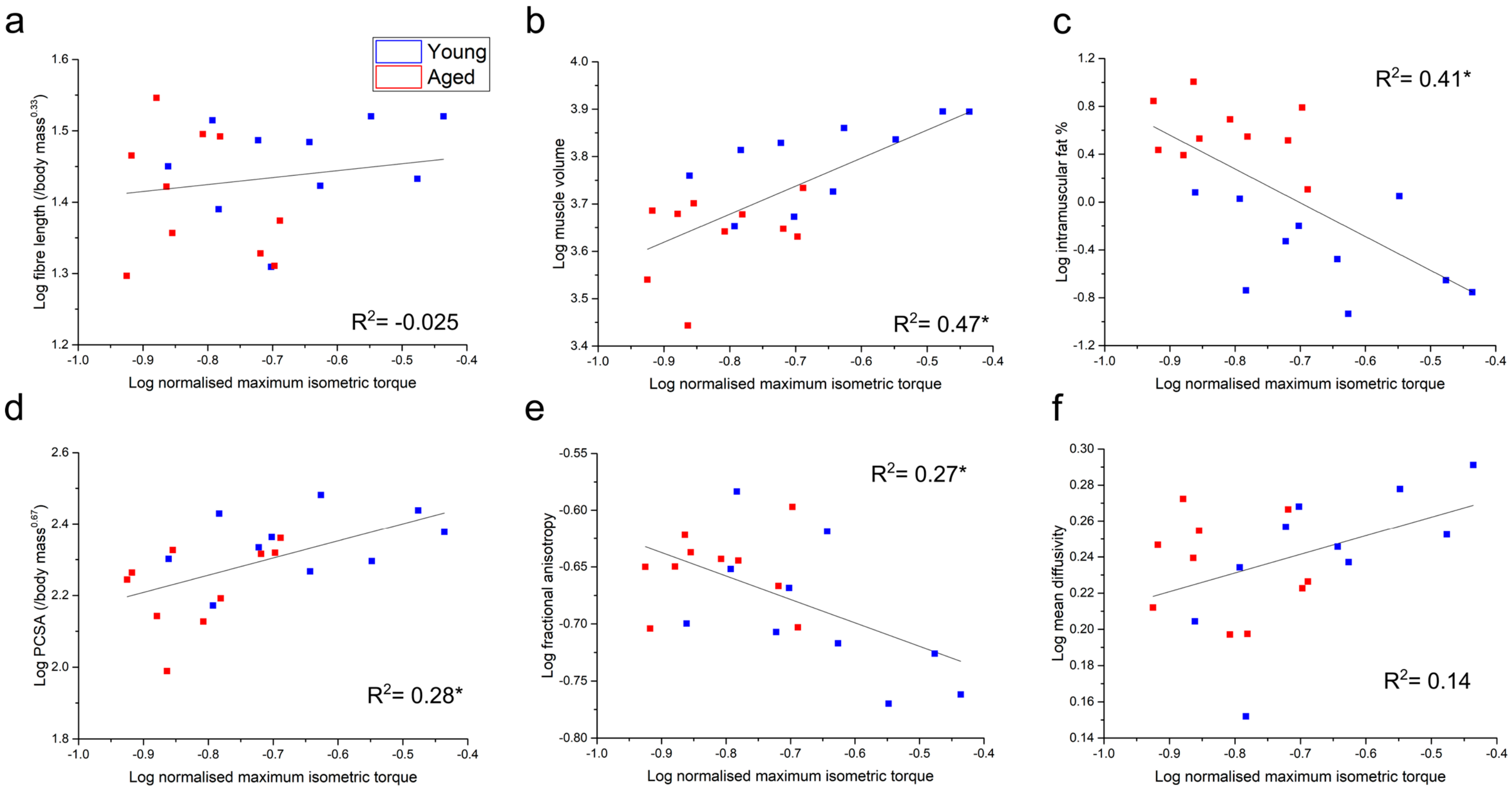

Figure S3. Linear relationships between maximum isometric knee extensor torque and fibre length (a), muscle volume (b), PCSA (c), intramuscular fat % (d), fractional anisotropy (e) and mean diffusivity (f) of the knee extensor muscles across the two populations (young and aged). * indicates statistically significant relationships ($p < 0.05$).

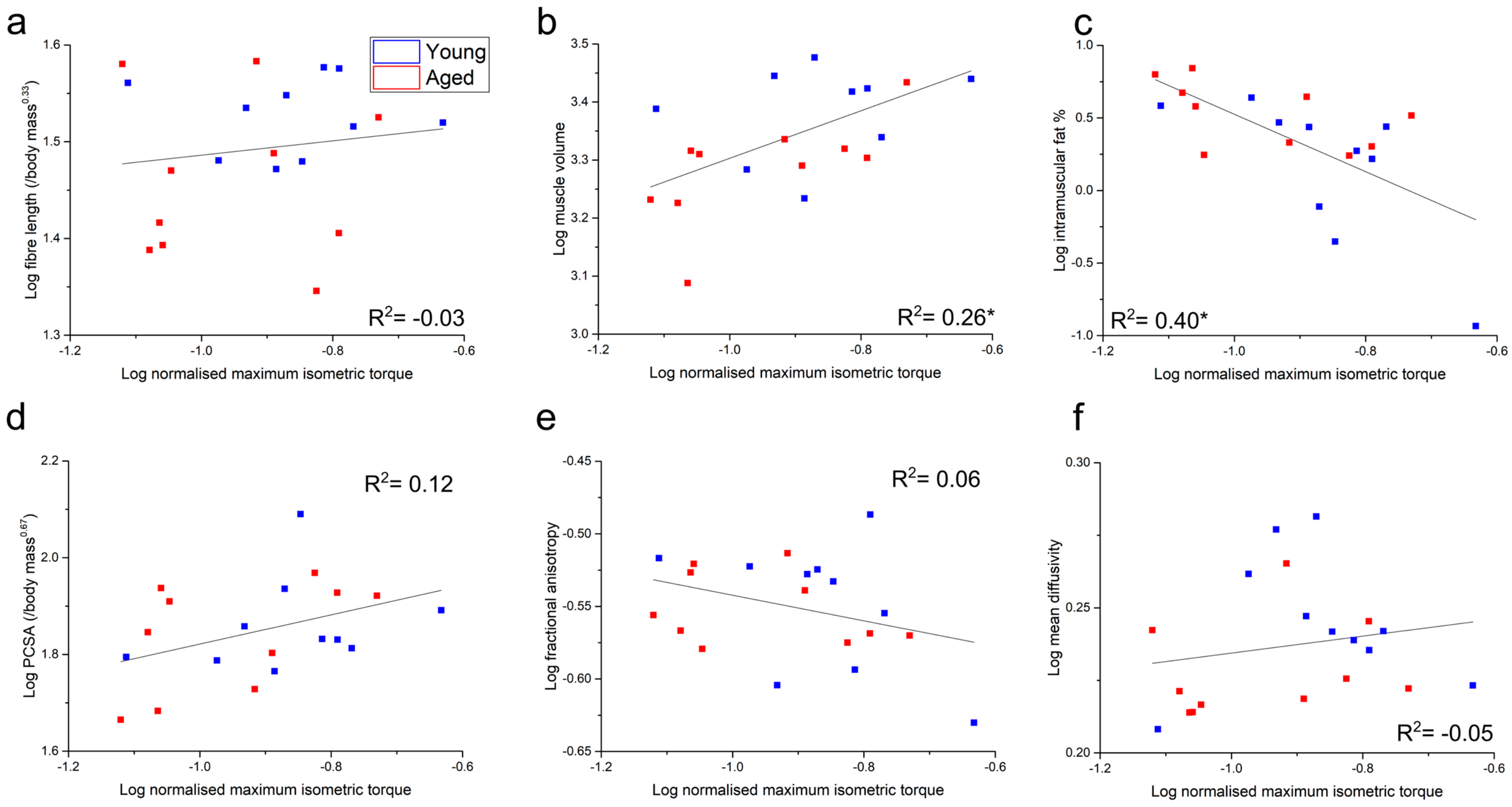

Figure S4. Linear relationships between maximum isometric knee flexor torque and fibre length (a), muscle volume (b), PCSA (c), intramuscular fat % (d), fractional anisotropy (e) and mean diffusivity (f) of the knee flexor muscles across the two populations (young and aged). * indicates statistically significant relationships ($p < 0.05$).

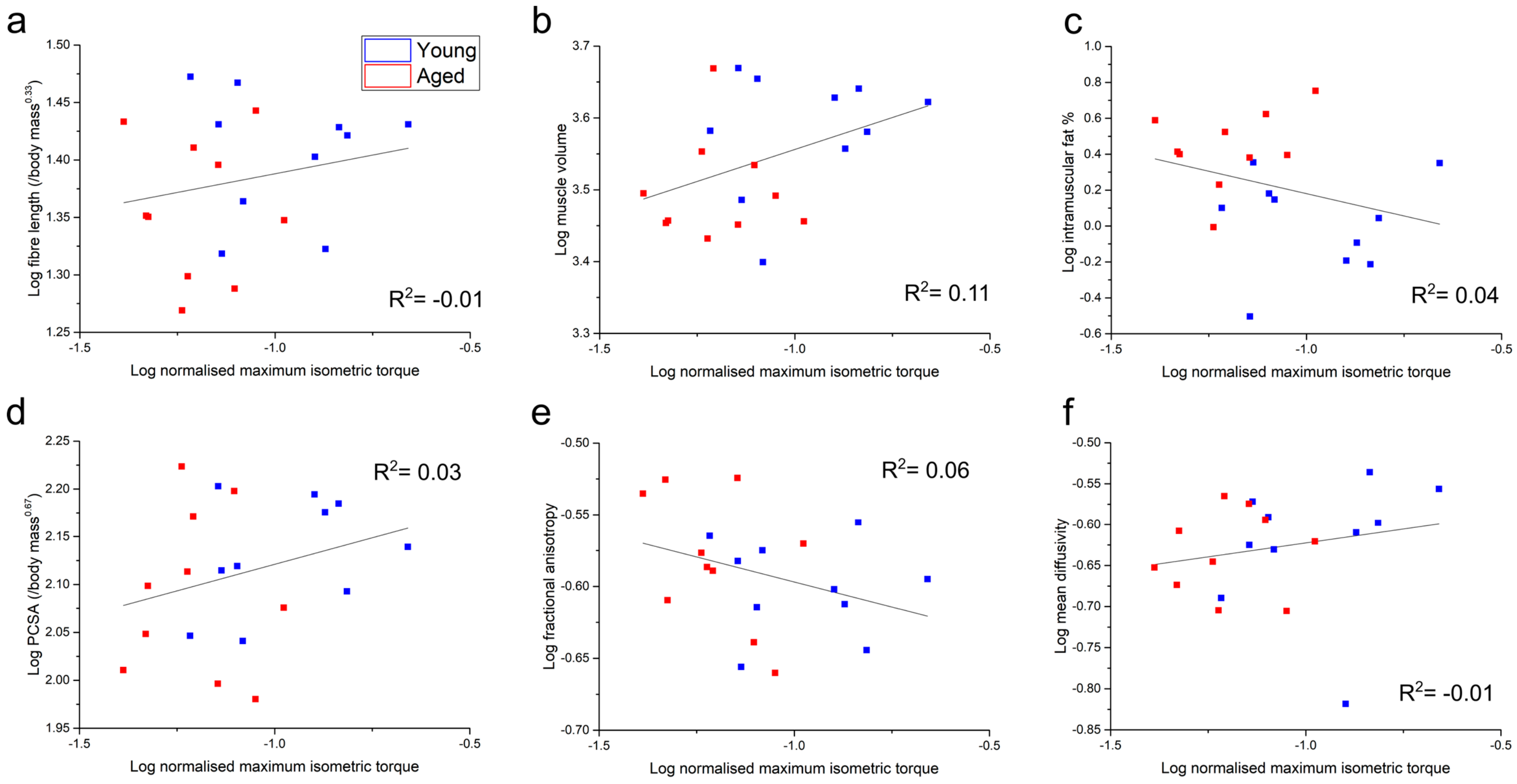

Figure S5. Linear relationships between maximum isometric ankle plantarflexor torque and fibre length (a), muscle volume (b), PCSA (c), intramuscular fat % (d), fractional anisotropy (e) and mean diffusivity (f) of the ankle plantarflexor muscles across the two populations (young and aged). * indicates statistically significant relationships (p < 0.05).

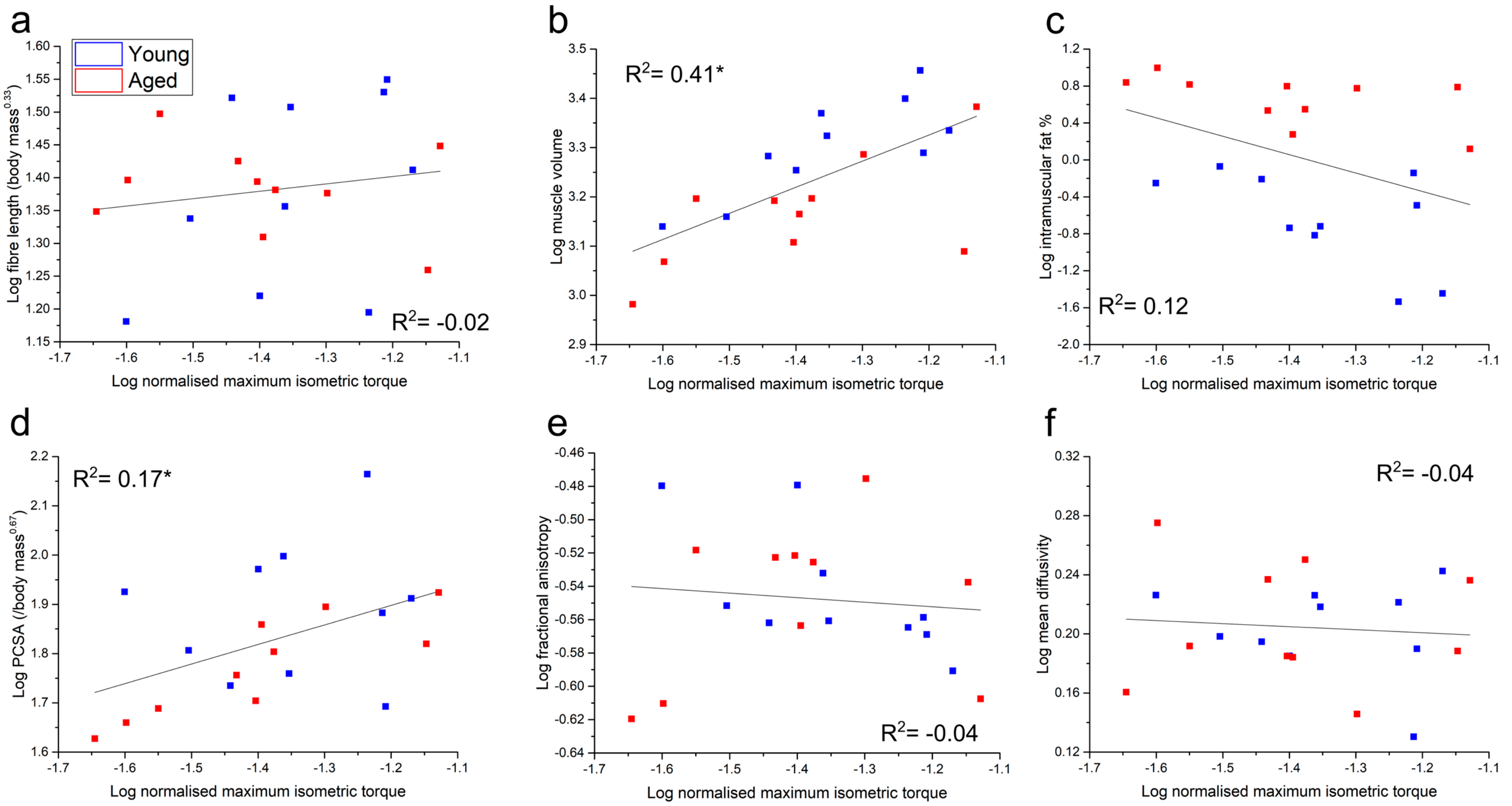

Figure S6. Linear relationships between maximum isometric ankle dorsiflexor torque and fibre length (a), muscle volume (b), PCSA (c), intramuscular fat % (d), fractional anisotropy (e) and mean diffusivity (f) of the ankle dorsiflexor muscles across the two populations (young and aged). * indicates statistically significant relationships ($p < 0.05$).

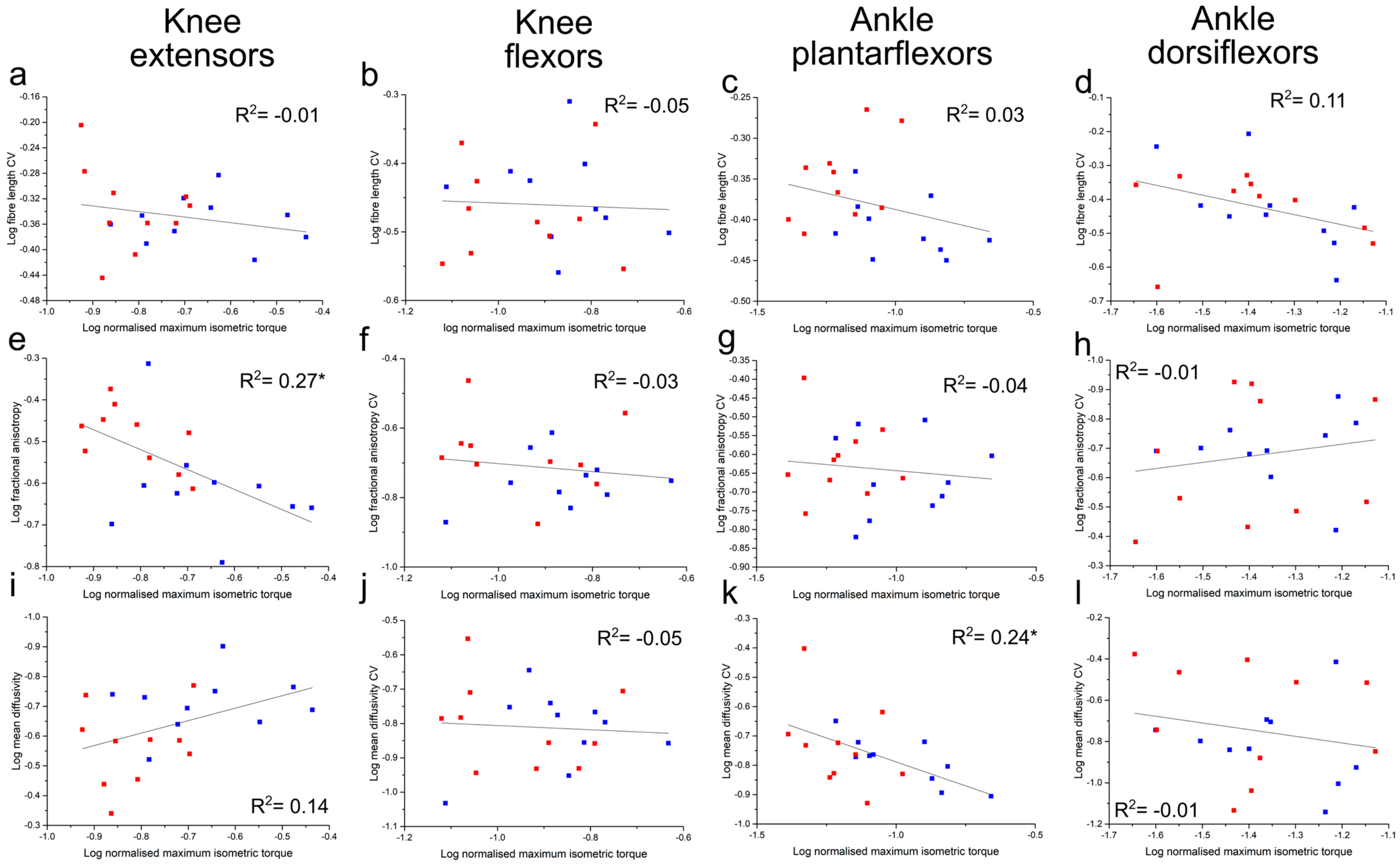

Figure S7. Linear relationships between maximum isometric torque and the coefficients of variation (CV; a quantification of heterogeneity) in fibre length (a-d), fractional anisotropy (e-h) and mean diffusivity (f-l) of the knee extensor, knee flexor, ankle plantarflexor and ankle dorsiflexor muscles across the two populations (young and aged). Significant relationships were found between FA CV and knee extensor torque (e) and between Lf CV and ankle dorsiflexor torque (d). * indicates statistically significant relationships ($p < 0.05$).

1. Charles J.P., Grant B., D'Aout K., Bates K.T. 2020 Subject-specific muscle properties from diffusion tensor imaging significantly improve the accuracy of musculoskeletal models. *J Anat* **237**(5), 941-959. (doi:10.1111/joa.13261).
